# Testing the Limits of a Portable NIR Spectrometer: Content Uniformity of Complex Powder Mixtures Followed by Calibration Transfer for In-Line Blend Monitoring

**DOI:** 10.3390/molecules26041129

**Published:** 2021-02-20

**Authors:** Tibor Casian, Alexandru Gavan, Sonia Iurian, Alina Porfire, Valentin Toma, Rares Stiufiuc, Ioan Tomuta

**Affiliations:** 1 Department of Pharmaceutical Technology and Biopharmacy, Faculty of Pharmacy, “Iuliu Hatieganu” University of Medicine and Pharmacy, 400012 Cluj-Napoca, Romania; casian.tibor@umfcluj.ro (T.C.); sonia.iurian@umfcluj.ro (S.I.); aporfire@umfcluj.ro (A.P.); tomutaioan@umfcluj.ro (I.T.); 2 Department of Medical Devices, Faculty of Pharmacy, “Iuliu Hatieganu” University of Medicine and Pharmacy, 400349 Cluj-Napoca, Romania; 3 MedFuture Research Center for Advanced Medicine, “Iuliu Hațieganu” University of Medicine and Pharmacy, 400337 Cluj-Napoca, Romania; valentin.toma@umfcluj.ro (V.T.); Rares.Stiufiuc@umfcluj.ro (R.S.)

**Keywords:** portable NIR, PAT, blending, validation, multivariate calibration

## Abstract

(1) Background: Portable NIR spectrometers gain more and more ground in the field of Process Analytical Technology due to the easy on-site flexibility and interfacing versatility. These advantages that originate from the instrument miniaturization, also come with a downside with respect to performance compared to benchtop devices. The objective of this work was to evaluate the performance of MicroNIR in a pharmaceutical powder blend application, having three active ingredients and 5 excipients. (2) Methods: Spectral data was recorded in reflectance mode using static and dynamic acquisition, on calibration set samples developed using an experimental design. (3) Results: The developed method accurately predicted the content uniformity of these complex mixtures, moreover it was validated in the entire calibration range using ±10% acceptance limits. With respect to at-line prediction, the method presented lower performance compared to a previously studied benchtop spectrometer. Regarding the in-line monitoring of the blending process, it was shown that the spectral variability-induced by dynamic acquisition could be efficiently managed using spectral pre-processing. (4) Conclusions: The in-line process monitoring resulted in accurate concentration profiles, highlighting differences in the mixing behaviour of the investigated ingredients. For the low dose component homogeneity was not reached due to an inefficient dispersive mixing.

## 1. Introduction

Blending can be considered as a critical processing step in pharmaceutical manufacturing as it directly influences the end product’s content uniformity [1]. This unit operation is applied for particulate systems such as powders, granules, capsules and tablets. From an economical perspective, avoiding out-of-specification batches and optimization of processing time are beneficial for the manufacturer [2]. Having a Process Analytical Technology (PAT) tool available for rapid product characterization, can guide the formulation and process development, scale up and detect process offsets during long term production, as a result of input variability. 

Content uniformity characterization is possible through off-line, at-line, on-line or in-line methods, the difference between measurements being resumed to the sample analysis location. For off-line and at-line measurements samples are removed from the process stream and are analysed at a remote location or in the near proximity of the process. On-line measurements involve the use of samples that are diverted from the main process in order to be analysed, whereas in-line measurements use invasive or noninvasive methods for direct analysis of samples without any rerouting from the process stream [3]. At-line prediction of product composition can be achieved using multivariate calibration procedure, whereas for real time process monitoring, there are several strategies which can be divided into qualitative (calibration free) and quantitative (calibration) methods [4,5]. 

Qualitative monitoring methods rely on assessing the similarity of recorded data with a target values through several methods: k-nearest neighbours (kNN), principal component analysis (PCA), soft independent modelling of class analogy (SIMCA), Euclidean distance, Discriminant Analysis; being frequently applied in early development [6]. PCA can be efficient in evaluating content uniformity, in this case relying on the score vs. concentration relationship and accurate loading estimation [7,8]. 

Calibration free, moving-block based methods, rely on estimating the standard deviation in spectra and assuming homogeneity under certain values. The main disadvantage of this approach is that deviations from declared content may not be detected. Additionally, they have a limited applicability in case of mixtures having components of interest with a reduced contribution to the spectral variability. The moving block standard deviation (MBSD) method can be customized to track peak height, area, or regions from spectral data which contain the desired features [1]. Its implementation for low dose constituents is accepted in the presence of strong and characteristic spectral features and if the spectral similarity of other constituents is increased [6]. 

Quantitative methods enable blend homogeneity evaluation and also confirm the presence of declared active pharmaceutical ingredient (API) content, reducing the risk of false predictions compared to qualitative methods. Such methods offer results expressed in the form of concentration profiles, and require extensive calibration to offer slightly biased predictions. 

There are numerous examples in literature demonstrating the implementation of PAT tools for the blending process, considering both batch and continuous manufacturing. Jarvinen et al. applied near Infrared spectroscopy (NIR) to monitor a continuous blending and tableting line. Calibration was done by recording spectra of various mixtures under dynamic acquisition mode through a sapphire window under the discharge chute of the continuous blender [9]. Li et al. used a calibration free semi quantitative NIR method to evaluate blend and content uniformity [8]. Some studies suggest the need for multipoint measurement systems for accurate estimation of blend homogeneity [6]. Vanarase et al. implemented a multipoint NIR system for continuous blending process. By measuring the velocity of the material it was possible to evaluate the number of scans representing the mass of one unit dose [10]. 

Blend uniformity tracking can also be successfully implemented during the tableting process by integrating a NIR probe into the feed frame [11]. This approach is useful for detecting segregation phenomenon [12].

One factor influencing accuracy of predictions during in-line measurements is offered by the moving dynamics of the particles, being influenced by powder speed and filling level. Within a continuous blending case study, Martinez et al. highlighted the sensitivity of partial least squares (PLS) models to flow dynamics, rotation speed, feeding rate of the material, making necessary the inclusion of such variability into the model if accurate predictions are desired. A qualitative monitoring approach was found appropriate in this case [2]. 

The vast majority of quantitative NIR applications rely on the use of benchtop devices, the performance of such equipment being well documented in the scientific literature. In the case of portable systems, the top applications seem to be associated with raw material and product identification. Their lower performance in quantitative analysis can be linked to the limited spectral range, lower resolution, worse signal to noise ratio and to the available recording configuration [13,14].

Using the MicroNIR spectrometer, process analytical solution could be implemented in a diverse range of applications. In our previous work, we accurately predicted the moisture evolution during fluid bed granulation process, having a robust prediction to changes in processing conditions and raw material characteristics [15,16]. Lee et al. successfully monitored the particle size distribution variation of lactose from three component mixtures during the blending experiment [17]. Sierra-Vega et al. used MicroNIR to predict the API concentration from a three component mixture at the discharge chute of a blender, respectively at the tablet feed frame. Specific absorption bands of the API had a major contribution to the accurate predictions [18]. Using MicroNIR, Tanimura et al. investigated the residence time distribution of an API in the tablet press feed frame in the case of a five component formulation. Predictive performance of the method was slightly affected only by paddle speed [19]. 

The major benefits associated with portable instruments come from the portability and their interfacing versatility, although the miniaturization comes with a price in terms of performance. Although it is considered that portable devices will play a key role in the implementation of PAT framework and pharmaceutical development, more performance evaluation studies are needed in this field [13].

To this respect there are several more recent studies oriented on the comparison of such instruments. Mayr et al. compared the performance of handheld and benchtop NIR devices, highlighting the importance of the recorded wavelength regions, sample presentation and analyte concentration [14]. Moreover, it was shown that the applied modelling approach can also have a great contribution to predictive power. In the prediction of moisture from plant matrices, handheld spectrometers could obtain comparable performance to benchtop devices only with non-linear modelling [20]. Ciza et al. developed similar NIR methods for the characterization of antimalarial medicine using both type of instruments [21]. 

The objective of this study was to evaluate the performance of a portable NIR spectrometer (MicroNIR PAT-U, Viavi Solutions, Wichita, KS, USA) by considering a complex powder mixture with three APIs and five excipients. In the case of this specific powder mixture, we previously demonstrated that the successful implementation of a NIR method strongly depended on the calibration set construction and on the orthogonality transfer form concentration space (powder composition) to spectral space. Using a bench-top NIR spectrometer in transmission configuration, the method presented an excellent performance and could be validated using ±5% and ±10% acceptability limits for the APIs found in higher, respectively lower concentration [22]. This work comes to extend previous results, by applying the previously prepared calibration set to train models using spectra recorded on a portable NIR spectrometer in reflectance mode. Additionally, the performance of this process-suited NIR instrument was evaluated in terms of process monitoring and tracking ability of both high and low-dose constituents during the pharmaceutical development of a drug product. 

## 2. Results and Discussions

### 2.1. Calibration Model Development and Validation for At-Line Content Uniformity Prediction

Two pre-processing methods worked better for data recorded with MicroNIR, namely the standard normal variate (SNV) and the second derivative (SD). The increased concentration of paracetamol and ibuprofen was reflected in the performance parameters of PLS models, showing good predictive capacity (Q^2^>0.9) with reduced cross-validation errors, achieved through a reduced number of components. The lower content in caffeine, slightly impacted the prediction ability, however, a good performance was still achieved for at-line characterization (Table 1). 

**Figure 1:**
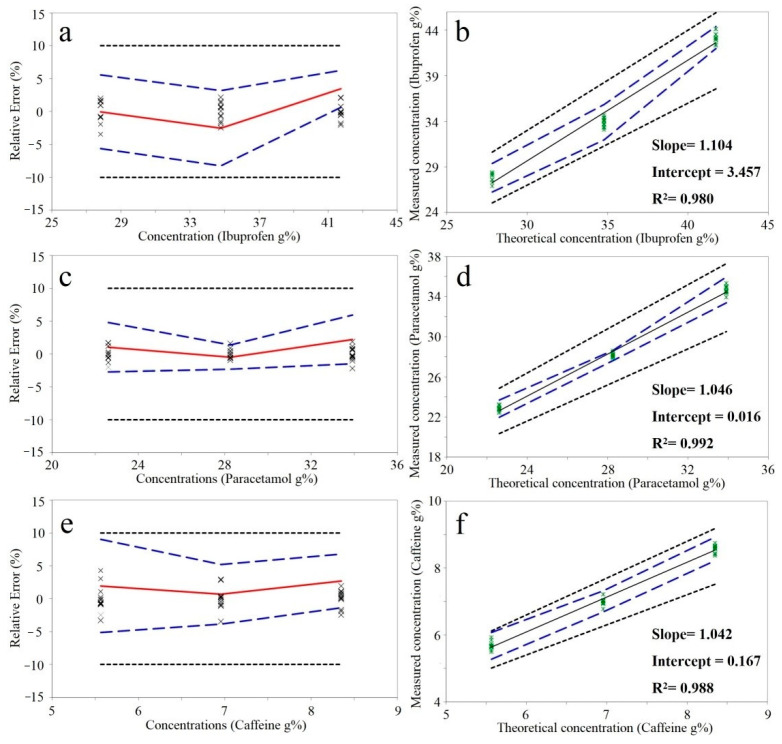
Accuracy (left) and linearity profiles (right) obtained for the at-line quantification of ibuprofen (**a**,**b**), paracetamol (**c**,**d**) and caffeine (**e**,**f**).

The at-line NIR method was validated using the strategy proposed by Hubert et al. [23]. According to the results, the method is capable of predicting the API content with an acceptable recovery and precision (Table 2). The calculated relative tolerance limits were included in the ±10% acceptability interval for all three APIs (Figure 1). The differences between real and predicted concentrations of future samples will be included in the computed tolerance limits with a 95% probability. 

Results published in our previous study, highlighted improved validation results when a bench top device on the identical calibration/validation sets. The relative tolerance limits for ibuprofen and paracetamol were included in ±5%, whereas for caffeine in ±10% limits. The technical characteristics of the used spectrometers are presented in Table 3. 

The similar performance for caffeine can be explained considering that the most characteristic absorption bands in the NIR spectra are recorded by both benchtop and portable devices. A similar result was obtained for this active ingredient by Mayr et al. [14]. 

In comparison with the benchtop device, unexpectedly, the lower performance was obtained for the large dose active ingredients. Although the most important absorption bands were included in the acquisition domain of MicroNIR, most certainly the spectral resolution and recording configuration had an impact on prediction performance. Transmission spectra recorded by the benchtop device is more representative compared to the reflectance spectra recorded with MicroNIR. Overall, for all the APIs the accuracy limits were included in the ±10%, thus making the portable instrument suitable for at-line characterization.

In another study, Ciza et al. obtained similar relative tolerance limits after validating two NIR methods, using spectra recorded with benchtop and handheld instruments. In this case, both methods were implemented in transmission configuration [21]. 

Dotted black line: acceptability limits; Dashed blue line: upper and lower relative 95%-β-expectation tolerance limits; Continuous red line: relative bias; X: relative back-calculated concentrations of validation samples.

### 2.2. Impact of Acquisition Mode on Spectral Variability

MicroNIR is a fiber-optic free device based on linear variable filter technology as the dispersing element [16]. The absence of moving parts makes it robust for in-line monitoring, by being less sensitive to process-induced shocks.

**Figure 2:**
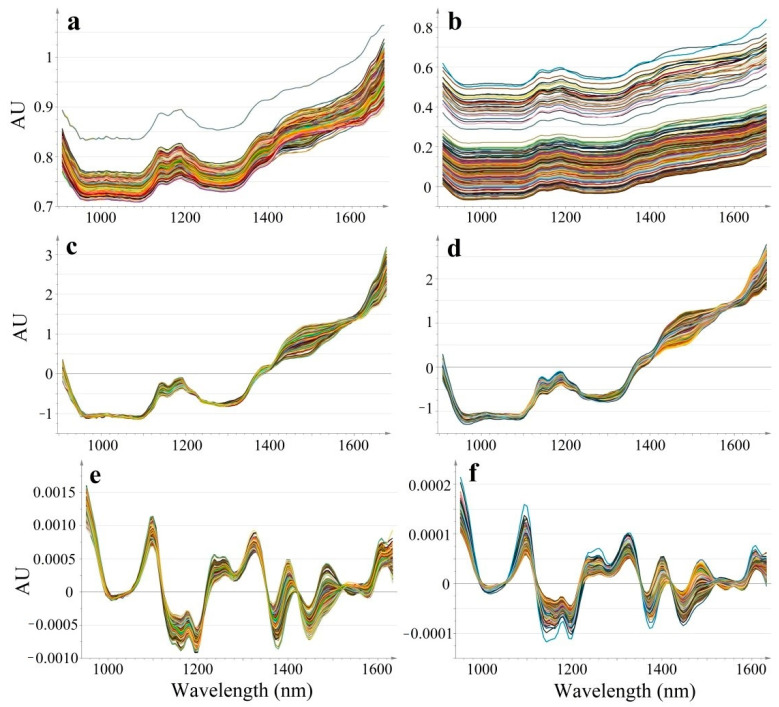
Raw and pre-processed spectral data recorded on calibration samples using static (at-line) and dynamic (in-line) acquisition modes (At-line: Raw (**a**), SNV (**c**), SD (**e**); In-line: Raw (**b**), SNV (**d**), SD (**f**)).

**Figure 3:**
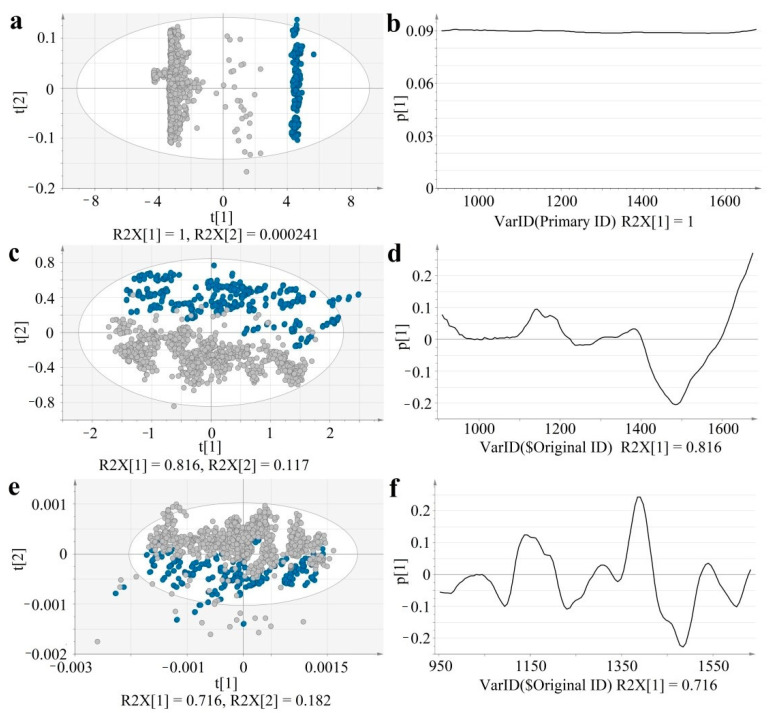
Score scatter and loading line plots of PCA models built on Raw (**a**,**b**), SNV (**c**,**d**), SD (**e**,**f**) processed at-line and in-line data (blue: at-line data; grey: in-line data).

Dynamic acquisition produced a higher baseline shift compared to static spectral recording, however spectral pre-processing methods efficiently reduced the differences (Figure 2). For an easier visualization and numerical interpretation of spectral variability, PCA models were computed for each type of data (raw, SNV, SD). The score and loading plots of the computed PCA models are shown in Figure 3.

In the case of raw data, the first principal component separates the two acquisition modes, representing the major variability in spectral data (R^2^X[1] = 1). The corresponding loading of these first principal components, shows a positive value throughout the entire domain, suggesting that the acquisition mode-related baseline-shift strongly dominates over the concentration-related variability. Thus, raw data is not efficient for predicting API content.

Salub et al. compared offline with inline spectra, showing that the baseline shift differences could be minimized through appropriate pre-processing methods. Highly predictive PLS models could be developed, also showing robustness to bin size and revolution speed changes [24].

Spectral pre-processing efficiently reduced sample presentation-related spectral variability, for both SNV and derivative data the separation of the groups occurs along the t[2] axis, representing the second principal component. The acquisition mode-related variability dropped in case of SNV data to R^2^X[2] = 0.117 and SD data to R^2^X[2] = 0.182, whereas the major variability captured by t[1] reflects composition differences. The p[1] loading vectors of SNV and SD data highlight characteristic peaks.

t[1]–first principal component; t[2]–second principal component; R2X–explained variability; p[1]–loading vector of the first principal component; Ellipse: Hotelling’s T2 (95%).

### 2.3. Monitoring Model Development

Considering the applicability of qualitative and moving-block monitoring strategies are not suitable due to the low concentration of caffeine, only quantitative modelling approaches were tested.

Calibration model development has to include the expected range of variability that could occur during a real blending process. To this respect, three different approaches were tested for quantitative model development, using either at-line or in-line collected spectra and the combination of both. Model performance parameters of the best models are presented in Table 1.

Both in-line recorded and combined spectra lead to a good predictive capacity (Q^2^ > 0.9) with reduced cross-validation errors, achieved through a reduced number of components. For all the tested modelling approaches, the prediction of paracetamol content outperformed ibuprofen results.

For caffeine, higher number of PLS components were fitted to the model, especially under dynamic conditions, as the reduced spectral contribution of this formulation constituent is more prone to be affected by the undesired spectral variability-induced by sample dynamics. Static spectral acquisition exceeded dynamic mode in terms of Q^2^, having above 0.9 value.

The resolution used to record spectral data is an important parameter as it influences both noise and acquisition time. A fast acquisition time and good signal to noise ratio is essential for process monitoring and can be obtained through low resolution measurements. However, using a too low resolution may pose robustness issues considering the amount of spectral information. This can also affect the quality of predictions where multiple constituents are varied in the blends (i.e., three APIs) [25]. MicroNIR is a process compatible instrument, thus working with such a complex mixture presenting multiple APIs certainly influenced the quality of predictions.

### 2.4. Application of the Models for In-Line Monitoring of a Blending Experiment

**Figure 4:**
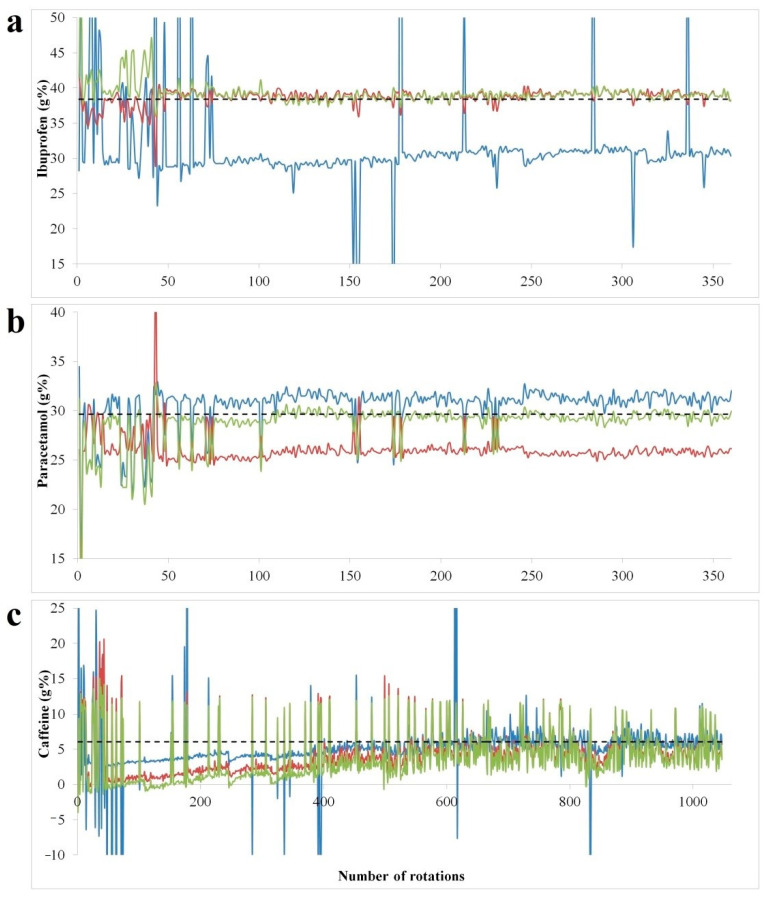
Predicted concentrations of ibuprofen (**a**), paracetamol (**b**) and caffeine (**c**) in function of number of rotations. Blue line: at-line model; red line: in-line model; green line: at-line+ in-line model; dotted black line: target concentration confirmed by HPLC.

As the root mean square cross-validation error (RMSECV) relates to left out data during model building procedure, the in-line testing of the developed models is mandatory for a true performance evaluation. The spectral data acquired during the blending experiment was transformed into concentration profiles using models developed with at-line; in-line and combined calibration data (Figure 4). Building individual mixing profiles of formulation constituents is beneficial for in depth understanding of blending, segregation and process endpoint identification [26]. The target concentrations reflecting the reach of homogeneity was confirmed through the reference method.

The concentration profiles show a highly spiked profile in the initial part of the blending process, with highly variable concentrations which decrease in amplitude and stabilize as homogeneity is reached. In the case of ibuprofen, the at-line model shows an under-prediction from the initial points of the blending run, whereas the in-line and combined model offers good predictions, close to reference method results.

For paracetamol the combined model has the best predictive performance, the at-line shows a positive bias, whereas in-line data under-predicts the concentration. In terms of paracetamol and ibuprofen content, blend homogeneity was reached under 300 rotations of the blender, whereas for caffeine the highly spiked profile suggested the lack of an efficient dispersive mixing.

In the case of caffeine, the at-line model had the best predictive performance, however the highly spiked profile was maintained up to 1000 revolutions. The recovery against reference method results was found acceptable for all APIs (Table 4). For caffeine the same concentration variability was observed by high performance liquid chromatography (HPLC) (6.077 ± 1.078), confirming the reduced efficiency of the blending process itself. In this case, convective blenders could lead to a more efficient distribution of caffeine in the mixture [27].

The difference in prediction quality of the models built using static and dynamic acquisition modes of calibration sets seems to be API-related. The spectral contribution of the target component in relation to other excipients and its robustness to process-induced variability can be a determining factor. According to the literature data, good models could be obtained using both recording conditions [28].

Blend homogeneity is dependent on the amount of API in the formulation and blending time. Discrete Element Modelling simulations identified the combination of high API amounts and low blending time in order to achieve optimal homogeneity [27]. Martinez et al. also highlighted the dependency of time needed to reach steady state concentration and API concentration. APIs with high mass fraction in the formulation reach in shorter time homogeneity [28]. These observations are confirmed by the experimental results of the current study.

## 3. Materials and Methods

### 3.1. Materials

The powder mixtures used for calibration and validation purposes contained: ibuprofen (IOL, Barnala, India), paracetamol (Hebei Jiheng, Hengshui, China), caffeine (Siegfried, Zofingen, Switzerland), microcrystalline cellulose (Sigachi, Hyderabad, India) as a diluent, sodium starch glycolate (Blanver, Sao Paulo, Brazil) as disintegrant, hydroxypropyl-methylcellulose (Colorcon, Stoughton, WI, USA) as binder, colloidal silicium dioxide (RhomPharma Polymers, Darmstadt, Germany) as glidant, magnesium stearate (Union Derivan, Barcelona, Spain) as lubricant.

### 3.2. Calibration Set Development

The calibration set composition was constructed using an experimental design based methodology, with a D-optimal design, resulting in a number of 31 different formulations. The concentration of the three active ingredients was varied in the range of 80-90-100-110-120%, having as center points 34.78% ibuprofen, 28.26% paracetamol and 6.95% caffeine. The amount of diluent was calculated for each formulation to ensure identical tablet mass. The concentration of all other excipients was not varied. More information on the calibration set development and composition can be consulted in the previously published work [22]. The quantitative composition of the formulation could not be divulged.

### 3.3. Spectral Acquisition

Diffuse reflectance spectra were recorded with a MicroNIR PAT-U spectrometer (Viavi Solution, San Jose, CA, USA) in the 950-1650 nm region and a resolution of 6.2 nm. Before starting the measurements, an external white reference (Spectralon) and a black reference were scanned and repeated every 15 min. Spectral data was recorded in static conditions (at-line) under controlled illumination conditions respecting a 3 mm distance from the probe to the sample.

**Figure 5:**
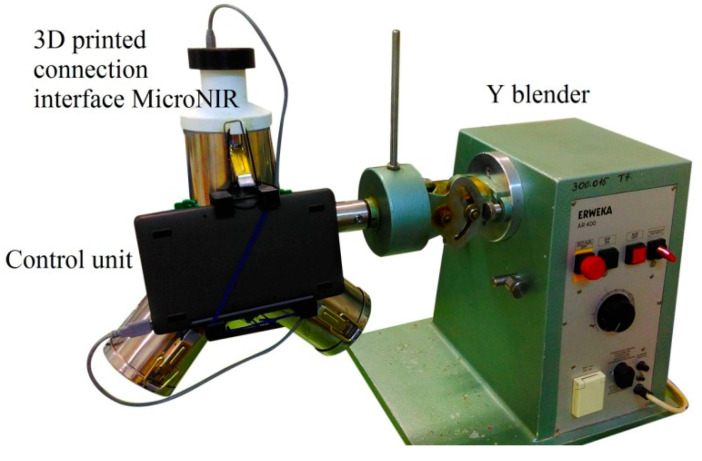
Experimental setup for in-line monitoring of the blending process.

The reduced size and weight of the instrument simplified the interfacing procedure. The handheld spectrometer was attached to the filling port of the Y blender through a 3D printed interfacing accessory, thus the recording of spectra was done noninvasively without interrupting the process (Figure 5). This setup was used for both in-line acquisition of calibration spectra and real time monitoring of a blending experiment.

Dynamic spectral acquisition was done with the in-line setup by placing a sample holder in the region of the interfacing port, to reduce the need for excessive powder material. For both acquisition modes the same powder blends were used. The distance from the sample holder to the NIR instrument enabled continuous flowing of the material, thus ensuring the recording of spectra from different parts with each revolution. Spectral data were recorded when the sensor was covered in powder, with the NIR facing a downward position. For in-line monitoring of the blending experiment the same conditions were used, one spectrum was recorded at each full rotation. For in-line experiments, a 7 msec acquisition time and integration of 200 spectra were selected.

For both static and dynamic acquisition modes, a number of 21 spectra were recorded for each of the 31 calibration set formulations, resulting in a total of 651 spectra.

### 3.4. Modelling Approaches

For the at-line prediction of powder content uniformity, PLS calibration models were developed using as input the spectra recorded under static condition and having as output the API concentration. To enable in-line monitoring of the blending process, three calibration models were developed by considering for the training: spectral data recorded under static condition, dynamic condition and their combination.

Spectral pre-processing methods were tested for scattering and baseline shift reduction purposes. Derivatives were calculated using a quadratic order polynomial having 15 points in each model and a distance between each point equal to one. Moreover, in order to compensate for the edge effects, the first and last window size of 7 variables are left out empty by default.

Before fitting the model, X variables were centered and Y variables were scaled to unit variance. Model performance was evaluated in terms of the explained variability (R^2^), predictive ability (Q^2^) and the root mean square cross-validation error (RMSECV). The number of components was selected considering the variation of RMSECV, in order to avoid over fitting [29,30].

### 3.5. Reference Method

The reference method used for content uniformity testing was a HPLC method combined with UV detection (Agilent, Santa Clara, CA, USA). A mobile phase comprised of Phosporic acid (0.01N) and Acetonitrile was pumped through a C18 Gemini μm 100 × 3 mm 110 A stationary phase, thermostated at 40 °C, using a 1mL/min flow rate. Analyte detection was carried out at 225 nm for ibuprofen, respectively at 275 nm for caffeine and paracetamol.

### 3.6. Validation

The validation protocol was carried out using accuracy profiles, on three concentration levels, having 18 external prediction set batches prepared and analysed in three different days. A number of 2 batches (10 samples/batch) were analysed daily for each concentration level, resulting a total of 180 spectra. Validation results, namely trueness, accuracy, precision and linearity were calculated and compared to the previous results obtained using a bench-top instrument [22].

### 3.7. Blending Experiments

Blending experiments were performed with a lab scale Erweka Y mixer equipment (Erweka, Langen, Germany). 152 g ibuprofen, 113.20 g paracetamol, 27.80 g caffeine, 67.50 g microcrystalline cellulose, 24 g sodium starch glycolate, 11.50 g Methocel E5LV, 2 g colloidal silicium dioxide, 2 g magnesium stearate were weighed accurately and transferred in the same order into the blending recipient. The quantitative composition was changed compared to the center formulation for a better evaluation of instrument performance. This combination of concentrations was not found within the calibration samples.

## 4. Conclusions

The present study demonstrates the feasibility of MicroNIR for the characterization of complex mixtures with three APIs and five excipients. The at-line NIR method was validated on the entire calibration range, between 80% and 120%, using ±10% acceptability limits.

Comparing the performance with a previously studied benchtop instrument, a lower performance was obtained for ibuprofen and paracetamol. The improved results obtained with the benchtop spectrometer can be attributed to the more representative transmission spectra and to the improved spectral resolution.

The portable PAT instrument could be efficiently used for real time prediction of all three active ingredients’ concentrations, offering accurate predictions and good recovery against the reference method. In the current experimental setup, the homogeneity could be reached only in terms of ibuprofen and paracetamol, as the lack of an efficient dispersive contributor to the blending mechanism caused an inhomogeneity in caffeine content.

The implementation of such a monitoring method provides a real time overview of the blending process, showing its evolution through time, thus allowing an early observation of potential problems and their analysis, or the establishment of the process’ end point at the moment when the desired homogeneity has been reached. In this way, the use of MicroNIR enhances process understanding and control, assuring that the quality of the medicine gets not only to be tested, but built into the product.

Applying in-line techniques eases process optimization and scale up, demonstrating that portable spectrometers have the potential to deliver the desired results within the PAT framework for complex formulations.

In point of fact, the described PAT tool has been used for the pharmaceutical development of a drug product, which has already been approved by authorities and is currently manufactured at industrial scale.

## Figures and Tables

**Table 1:** PLS model characteristics for active ingredient quantification by MicroNIR.

Acquisition	API	Pre-Processing	Spectral Region nm	PLS Components	R^2^X	Q^2^	RMSEC	RMSECV
at-line	Ibuprofen	SNV	908.1–1676.2	4	0.911	0.957	1.108	1.118
	Paracetamol	SD	951.46–1632.84	5	0.992	0.984	0.554	0.558
	Caffeine	SNV	908.1–1676.2	6	0.996	0.911	0.315	0.319
in-line	Ibuprofen	SD	951.46–1632.84	4	0.976	0.929	1.434	1.439
	Paracetamol	SD		6	0.993	0.968	0.745	0.756
	Caffeine	SD		11	0.998	0.899	0.337	0.344
at-line + in-line	Ibuprofen	SD	951.46–1632.84	3	0.916	0.913	1.566	1.589
	Paracetamol	SD		5	0.963	0.945	0.984	1.004
	Caffeine	SD		9	0.997	0.870	0.387	0.390

Abbreviations: PLS—partial least squares, API—active pharmaceutical ingredient, RMSEC—root mean square error of calibration, RMSECV—root mean square error of cross-validation.

**Table 2:** Validation results for the NIR method developed for at-line quantification.

Concentration Level (g%)	Trueness		Precision		Accuracy	
	Relative Bias(%)	Recovery (%)	Repeatability (RSD %)	Intermediate Precision (RSD %)	Relative Tolerance Limits (%)	Tolerance Limits (mg/tablet)
**Ibuprofen**						
27.814	−0.045	99.955	1.190	1.821	[−5.644, 5.544]	[26.256, 29.371]
33.898	−2.540	97.460	0.844	1.597	[−8.252, 3.172]	[31.962, 35.835]
43.182	3.458	103.458	1.412	1.237	[0.649, 6.268]	[41.969, 44.396]
**Paracetamol**						
22.843	1.035	101.035	0.810	1.223	[−2.720, 4.791]	[21.985, 23.702]
28.131	−0.459	99.540	0.709	0.768	[−2.298, 1.379]	[27.613, 28.648]
34.668	2.228	102.228	0.739	1.198	[−1.475, 5.931]	[33.384, 35.952]
**Caffeine**						
5.672	1.932	101.932	1.510	2.303	[−5.147, 9.012]	[5.270, 6.074]
7.003	0.682	100.682	1.520	1.784	[−3.846, 5.211]	[6.686, 7.320]
8.573	2.695	102.695	0.992	1.442	[−1.390, 6.782]	[8.222, 8.923]

**Table 3:** Technical specifications of the NIR spectrometers.

	Portable Spectrometer	Benchtop Spectrometer
Device	MicroNIR PAT-U (Viavi Solutions, San Jose, CA, USA)	MPA FT-NIR (Bruker Optics, Ettlingen, Germany)
	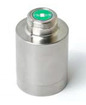	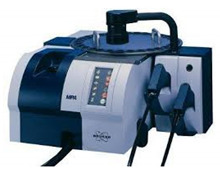
Spectral region	908–1676 nm (11,013–5966 cm^−1^)	12,497.2–4000 cm^−1^
Resolution	6.2 nm for 950–1650 nm	4 cm^−1^
Light source	Two integrated vacuum tungsten lamps	Tungsten-halogen lamp
Wavelength selection	linear variable filter	interferometer
Detector	128-pixel InGaAs photodiode array	InGaAs
Recording configuration	reflectance	transmission with rotating sample
Size	45 mm diameter × 69 mm tall; 368 g	570 mm length × 465 mm width 260 mm tall;

**Table 4:** Recovery of predicted API content against reference method.

Acquisition	API	Predicted C%	HPLC	Recovery
at-line	Ibuprofen	31.680 ± 6.101	38.412 ± 1.013	82.475
	Paracetamol	31.147 ± 0.478	29.644 ± 0.757	105.070
	Caffeine	6.256 ± 0.921	6.072 ± 1.078	103.045
in-line	Ibuprofen	38.951 ± 0.558	38.412 ± 1.013	101.400
	Paracetamol	25.816 ± 0.280	29.644 ± 0.757	87.085
	Caffeine	5.358 ± 2.031	6.072 ± 1.078	88.249
at-line + in-line	Ibuprofen	38.995 ± 0.361	38.412 ± 1.013	101.520
	Paracetamol	29.369 ± 0.399	29.644 ± 0.757	99.074
	Caffeine	5.315 ± 2.410	6.072 ± 1.078	87.534

## Data Availability

Data sharing is not applicable to this article. The data presented in this study is available on request from the corresponding author. The data are not publicly available due to comercial reasons.

## Sample Availability

Samples of the compounds are not available from the authors.
